# Nanoscale microenvironment engineering for expanding human hair follicle stem cell and revealing their plasticity

**DOI:** 10.1186/s12951-021-00840-5

**Published:** 2021-03-31

**Authors:** Peng Chen, Feifei Zhang, Zhexiang Fan, Tianding Shen, Bingcheng Liu, Ruosi Chen, Qian Qu, Jin Wang, Yong Miao, Zhiqi Hu

**Affiliations:** Department of Plastic and Aesthetic Surgery, Nanfang Hospital, Southern Medical University, 1838 North Guangzhou Avenue, Guangzhou, 510515 Guangdong China

**Keywords:** Human hair follicle stem cells, Stem cell microenvironment, Layer-by-layer self-assembly, Regenerative medicine, Tissue engineering

## Abstract

**Background:**

Periodically regenerated hair follicles provide an excellent research model for studying tissue regeneration and stem cell homeostasis. Periodic activation and differentiation of hair follicle stem cells (HFSCs) fuel cyclical bouts of hair regeneration. HFSCs represent an excellent paradigm for studying tissue regeneration and somatic stem cell homeostasis. However, these crucial studies are hampered by the lack of a culture system able to stably expand human HFSCs and regulate their fate.

**Results:**

Here, we use layer-by-layer (LbL) self-assembly with gelatin/alginate to construct a nanoscale biomimetic extracellular matrix (ECM) for an HFSC population. The LbL coating provides ECM and mechanical support for individual cells, which helps to maintain the CD200^+^α6^+^ HFSC population to a certain extent. Addition of key signal molecules (FGF-7 and VEGF-A) simulates the minimum essential components of the stem cell microenvironment, thereby effectively and stably expanding HFSCs and maintaining the CD200^+^α6^+^ HFSC population. Subsequently, BMP2 loaded to the nanocoated layer, as a slow-release signal molecule, activates BMP signaling to regulate HFSCs’ fate in order to obtain a purified CD200^+^α6^+^ HFSC population.

**Conclusion:**

This system can minimize the microenvironment of HFSCs; thus, stably amplifying HFSCs and revealing their plasticity. Our study thus provides a new tool for studies of hair follicle reconstruction and stem cell homeostasis.

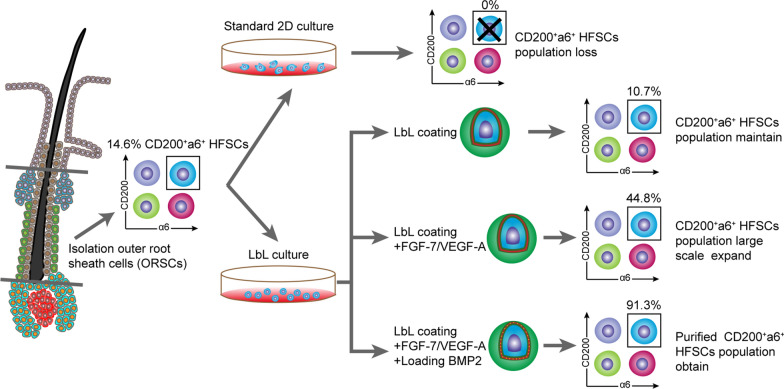

**Supplementary Information:**

The online version contains supplementary material available at 10.1186/s12951-021-00840-5.

## Background

Human adult stem cells (SCs) are critical for tissue homeostasis, repair, and regeneration [1, 2]. The distinct capabilities of SCs to self-renew and differentiate are central for the maintenance of organ homeostasis [3]. SCs are under strict homeostatic regulation, so that the system can adapt rapidly to interference to restore tissue function effectively. Human hair follicles are considered mini-organs with periodic regeneration and abundant sources, providing an attractive model for the tissue homeostasis and regeneration research.

The cyclical bouts of hair regeneration, growth (anagen), regression (catagen), and rest (telogen) are driven by the quiescent activation cycle of hair follicle stem cells (HFSCs) [4]. HFSCs are the key seed cells for tissue hair follicle reconstruction and provide an excellent paradigm for the study of somatic adult SC lineage [1]. Thus far, crucial studies have been hindered by the lack of a culture system able to expand human HFSCs and stably regulate their fate. Whereas various culture systems are dedicated to culturing HFSCs in vitro [5–7], there are no methods to maintain and expand true pluripotent human HFSCs in culture without fibroblast feeder cells, as well as in vitro methods for regulating the fate of human HFSCs and their progeny.

SCs are present in spatially distinct microenvironments, known as niches. The niches of SC comprise SCs themselves, neighboring cells, signals, and the extracellular matrix (ECM) [8]. In in vitro culture, SCs abandon their niche microenvironment and rapidly lose their distinct properties [9]. Previously, Chacón-Martínez et al. [10] established an in vitro culture system based on a 3D ECM environment and defined soluble factors that allowed murine HFSCs to expand and self-sustain on a long-term without heterologous cell types. However, whether the system is suitable for the culture of human HFSCs remains unknown. Besides, due to the hypoxia in the spheroid and contact inhibition in 3D culture, and the difficulty of digestion and passage, 3D culture for large-scale expansion of HFSCs is still hindered. Previously, we successfully constructed a 2D biomimetic microenvironment for mouse HFSCs that helped maintain the SC property of mouse HFSCs and regulate their fate to an extent [11]. Hence, exploring biomaterials to mimic SC microenvironments in 2D culture systems, amplify multipotent HFSCs, and regulate their fate, has become a promising solution for tissue homeostasis and regeneration studies.

Layer-by-layer (LbL) self-assembly as a thin film manufacturing technology for cell surface coating. This technique deposits a multilayer of nanocoating on the cell surface with oppositely charged polycationic and polyanion materials [12–14]. Due to the tunability and versatility of the coating layers, LbL cell coating has been successfully developed for various biomedical applications, such as cell-based biosensors [15], targeted gene therapy [16], cell/molecular delivery [17, 18], and tissue engineering [19]. LbL is different from other technologies for encapsulating cells into micro-hydrogels, which usually produce large cell aggregates and cannot control the mechanical properties of hydrogels. LbL can construct a biomimetic ECM for cells at the single-cell level and control the mechanical properties of the coating to provide mechanical support for the cells. Additionally, gelatin is a protein derivative, while alginate derived from algae. Because of their biodegradability and biocompatibility, these two biomaterials have been widely applied in ECM engineering [20–23]. Here, we applied LbL self-assembly with gelatin/alginate to coat human HFSCs for ECM engineering and maintain HFSC properties.

In vitro culture of human HFSCs usually requires extracting the outer root sheath cells (ORSCs) from the hair follicle, which are subsequently seeded on a feeder layer after fluorescence-activated cell sorting (FACS) of the bulge (CD200^+^α6^+^) HFSCs. This usually requires a large number of human hair follicles to obtain a tiny amount of purified CD200^+^α6^+^ HFSCs. Several studies have shown the plasticity of HFSCs even though the bulge normally produces hair germ; thus, the hair germ can replenish an empty bulge niche, which underscores their close relationship and capacity to interconvert when required [1, 24]. Moreover, studies have reported on the plasticity of mouse HFSCs in vitro with the addition of BMP or SHH inhibitors [10, 11]. However, no studies on the plasticity of human HFSCs in vitro exist. Furthermore, LbL self-assembly technology effectively regulates cell functions by loading certain bioactive macromolecules to the nanocoated layer [25, 26]. However, whether we can load active factors into LbL nanocoating for regulating the fate of human HFSCs and reveal their plasticity requires further verification.

Therefore, our study aimed to develop a novel 2D culture model by constructing a nanoscale microenvironment for HFSCs to expand human HFSCs and reveal their plasticity. To this end, we first isolated and extracted human HFSCs, and verified whether the LbL nanocoating of gelatin/alginate could be used for human HFSCs for constructing a nanoscale ECM. Then, we studied how LbL nanocoating based on single cells affects human HFSCs’ viability, proliferation, morphology, and SC properties. We also added defined factors to the medium to study the key factors required to reconstruct the essential components of the HFSC microenvironment. Finally, we loaded BMP2 to the nanocoated layer to study whether the nanocoated layer can act as a drug carrier for regulating HFSCs’ fate further to obtain CD200^+^α6^+^ HFSCs. Our 2D biomimetic culture system construct allowed human HFSCs to be expanded and maintained without feeder cells for tissue homeostasis and regeneration studies. Based on this system, we also revealed the plasticity of human HFSCs, providing a cellular mechanism for homeostatic regulation of the SC microenvironment.

## Results

### Isolation and culture of human HFSCs

**Fig. 1:**
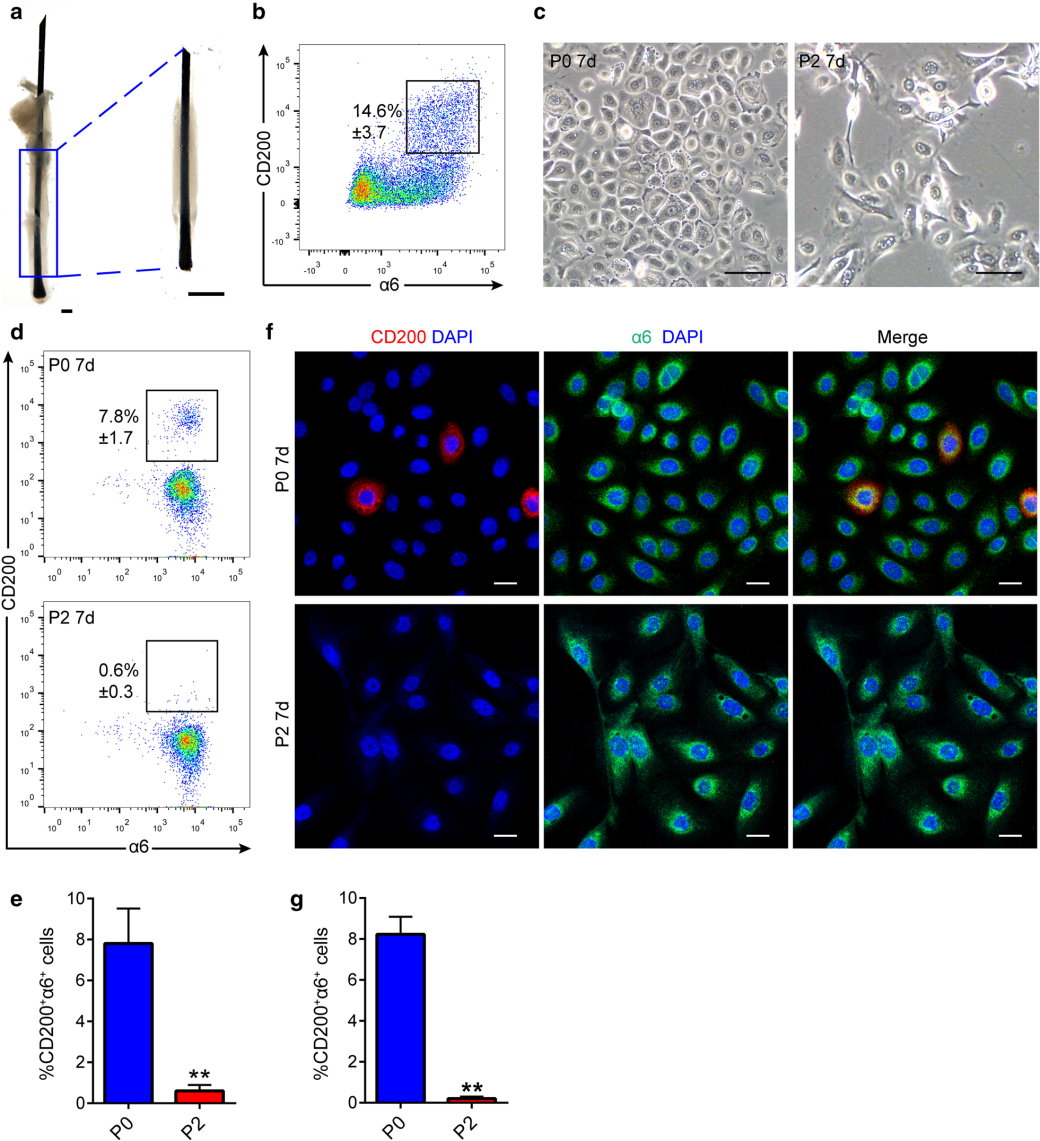
Culture of human HFSCs under standard 2D culture conditions. **a** After microscopic separation combined with enzyme digestion, the remaining hair follicle tissue containing the outer root sheath was obtained. Scale bars: 200 µm. **b** Flow cytometry analysis showed that the proportion of CD200^+^a6^+^ HFSCs in freshly isolated HFSCs from human hair follicles was 14.6% ± 3.7% (± SD). **c** The growth status of HFSCs was examined using a microscope in passage 0 (P0) and passage 2 (P2) under standard 2D culture conditions. Scale bars:200 µm. **d**, **e** Flow cytometry was performed on day 7 after culture. The results showed that CD200^+^a6^+^ HFSCs were significantly down-regulated under standard 2D culture conditions, and that no CD200^+^a6^+^ HFSCs were present in P2 (mean ± SD; n = 3; **p < 0.01, Student’s t-test). **f**, **g** Immunofluorescence staining was conducted on day 7 after culture, and the results showed that the proportion of CD200^+^a6^+^ cells decreased significantly under standard 2D culture conditions. No red-stained CD200^+^ HFSCs were present at P2 (mean ± SD; n = 4; **p < 0.01, Student’s t-test). DAPI (blue); CD200 (red); a6 (green); Scale bars: 20 µm

To construct a biomimetic microenvironment for HFSCs in vitro, we first isolated and cultured HFSCs. Freshly isolated ORSCs from human hair follicles contained 14.6 ± 3.7% (± SD) CD200^+^a6^+^ HFSCs **(**Fig. 1a, b). The isolated ORSC suspension was then cultured in standard 2D culture condition with keratinocyte growth medium (KGM) on the fibroblast feeder layer, which is widely used in keratinocyte culture [10, 27]. As shown in Fig. 1c, under this culture condition, the cell grew well at P0; however, the cell morphology changed and proliferation declined after passage P2, and cells could not be passaged. The cloning formation assay further confirmed that the proliferation of HFSCs at P2 was significantly lower than that at P0 (Additional file 1: Figure S1). Flow cytometry and immunofluorescence staining analysis showed that the CD200^+^α6^+^ HFSC population of the cells grown under these conditions was depleted after culture (Fig. 1d–g). These results indicate that under standard 2D culture conditions, HFSCs rapidly lose their proliferation ability and SC properties.

### LbL nanocoated HFSCs and their characterization in vitro

**Fig. 2:**
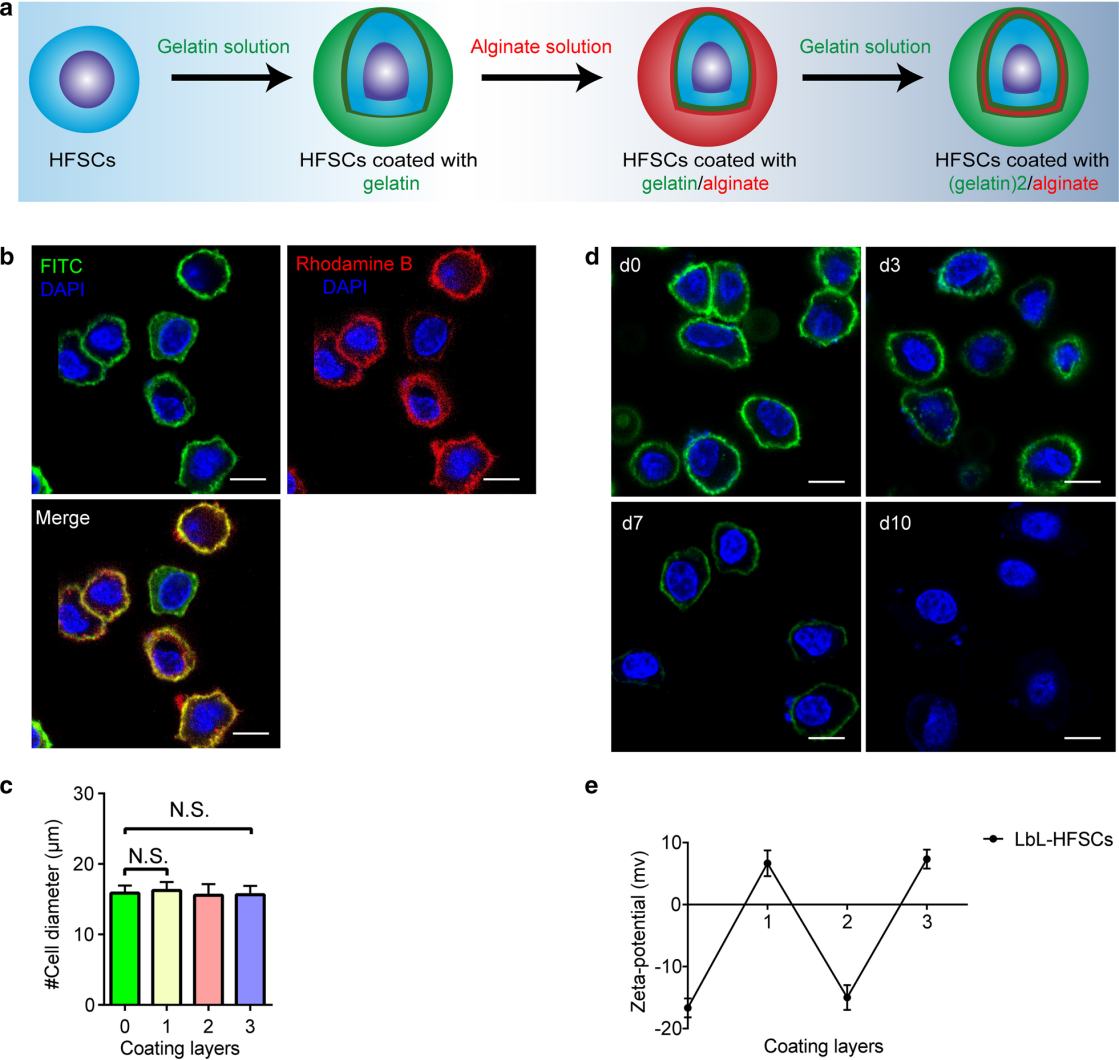
Generation of LbL-HFSCs based on the LbL cell nanocoating technique. **a** Schematic diagram of coating human HFSCs to construct LbL-HFSCs. **b** Images of confocal laser scanning microscopy (CLSM) show the HFSCs coated with gelatin-FITC (green)/alginate-rhodamine B (red). The cells are in suspension. DAPI (blue). Scale: 10 µm. **c** The cell diameter after different nanocoating layer was calculated using Image-Pro Plus 6.0 based on the images of CLSM (mean ± SD; n = 4; NS, not significant; *p* > 0.05, one-way ANOVA). **d** CLSM images displaying the existence of the biomaterials coated on the surface of the HFSCs by LBL. The cells are in suspension. DAPI (blue); Gelatin-FITC (green); scale: 10 µm. **e** Zeta-potential changes per cell surface coating layer (mean ± SD; n = 4): (0) uncoated HFSCs, (1) gelatin-coated HFSCs, (2) gelatin/alginate- coated HFSCs, (3) (gelatin) 2/alginate- coated HFSCs

ECM components are crucial in directing the proliferation, migration and polarity, differentiation, and status of SCs [28]. Some ECM components play an important role in ensuring the SCs remain undifferentiated [29–31]. Natural ECM polysaccharides and proteins can be used as scaffolds to create a natural ECM-like environment, which mimics the mechanical and physical properties required for the niches of SCs in vivo [32]. First, we used LbL nanocoating technology to coat human HFSCs with gelatin and alginate to construct a single-cell-based nano-scale ECM (Fig. 2a). Next, we used FITC-conjugated gelatin/rhodamine B-conjugated alginate to observe the LbL nanocoating on the surface of the HFSCs. From the results, each nanocoated layer displayed relevant green or red fluorescence (Fig. 2b). The cell size did not change significantly after each nanocoating step (Fig. 2c). In addition, the duration of the coating biomaterial on the cell surface was observed by coating HFSCs with (gelatin-FITC)-alginate-gelatin (Fig. 2d). The fluorescence intensity on the cell surface gradually decreased over time from 0 to 10 days, which indicated that the biomaterials were gradually degraded and could be maintained for about 7 days, thereby supplying adequate time for their applications and functions. Moreover, a zeta-potential analysis was performed to evaluate the potential cell changes in different layers of the nanocoating (Fig. 2e). The zeta potential varies with different levels of biomaterials. Oppositely charged polyelectrolytes are combined with each other and deposited. The results indicate that LbL nanocoating by gelatin/alginate was applied to human HFSCs successfully.

### LbL coating as a scaffold maintained the spheroid-like morphology of HFSCs without affecting cell viability and proliferation

**Fig. 3:**
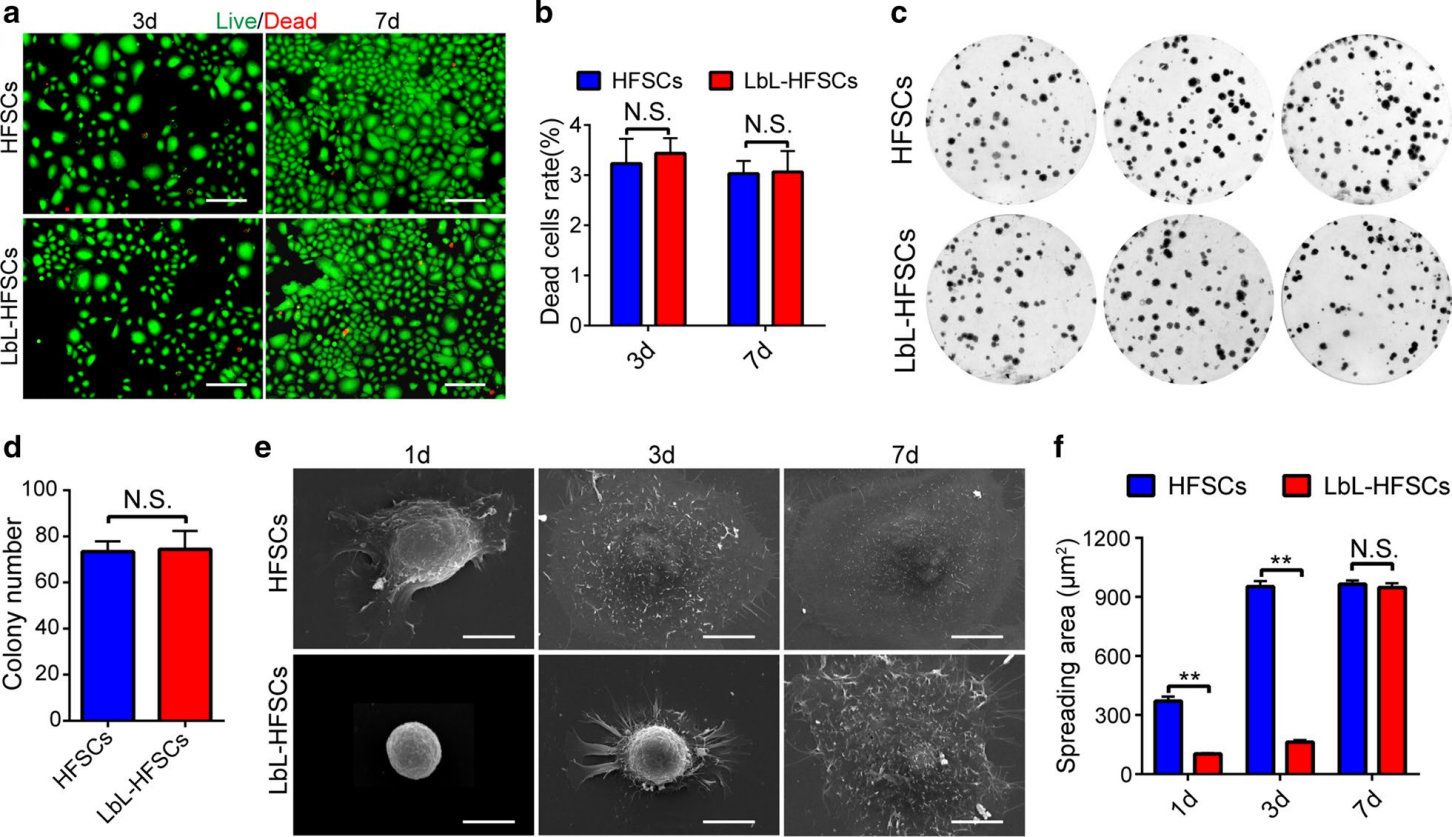
The effect of LBL coating on HFSCs’ viability, proliferation, or morphology. **a** Live/dead staining shows the dead cells in the uncoated HFSCs and LbL-coated HFSCs on days 3 and 7 of culture. Dead (red); Live (green); Scale bars: 100 µm. **b** There was no significant difference in the proportion of dead cells between uncoated HFSCs and LbL-HFSCs (mean ± SD; n = 4; NS, not significant; *p* > 0.05, Student’s t-test). **c** Colony formation assays detected the effect of LbL coating on the proliferation of HFSCs. **d** Number of clones between the uncoated HFSCs and LbL-coated HFSCs. No significant difference was observed (mean ± SD; n = 4; NS, not significant; *p* > 0.05, Student’s t-test). **e** The morphology of uncoated HFSCs and LbL-HFSCs on day 1, 3, and 7 was evaluated by SEM. Scale bars: 10 µm. **f** On days 1 and 3 after coating, the stretched area of the HFSCs was calculated using Image-Pro Plus 6.0 based on the SEM results. The stretched area of the LbL-coated HFSCs was lower than that of the uncoated HFSCs; however, on day 7, there was no significant difference (mean ± SD; n = 4; NS, not significant; *p* > 0.05, **p < 0.01, Student’s t-test)

After successfully using LbL to coat a single HFSC (with gelatin and alginate used to construct a biomimetic ECM), we examined how LbL nanocoating using gelatin/alginate affects HFSCs’ viability, proliferation, and morphology. On the third and seventh days after LbL nanocoating, live/dead staining results showed low cell death rates of untreated and LbL-treated HFSC. There was no significant difference between the two groups (*p* > 0.05; Fig. 3a, b). These data indicate that the nanoscale ECM produced by LbL cell nanocoating was not harmful to the viability of HFSCs. Immunostaining for Ki-67 was subsequently performed on days 3 and 7 after coating to evaluate the proliferation of HFSCs in the two groups (Fig. 3c). As shown in Fig. 3d, no significant difference was detected (*p* > 0.05). SEM analysis was carried out on both groups on days 1, 3, and 7 after LbL nanocoating to evaluate the cell morphology (Fig. 3e). The LbL-coated HFSCs exhibited a spheroid morphology at an early time point after coating, while those that were uncoated exhibited a broadly stretched morphology. However, on day 7 after coating, there was no significant difference between the two groups ((*p* > 0.05; Fig. 2f), indicating that LbL coating as a scaffold can provide mechanical support for HFSCs to morphologically display more in vivo-like 3D structures at an early time point after coating.

### ***LbL coating helped maintain the CD200***^+^***α6***^+^***HFSC population***

**Fig. 4:**
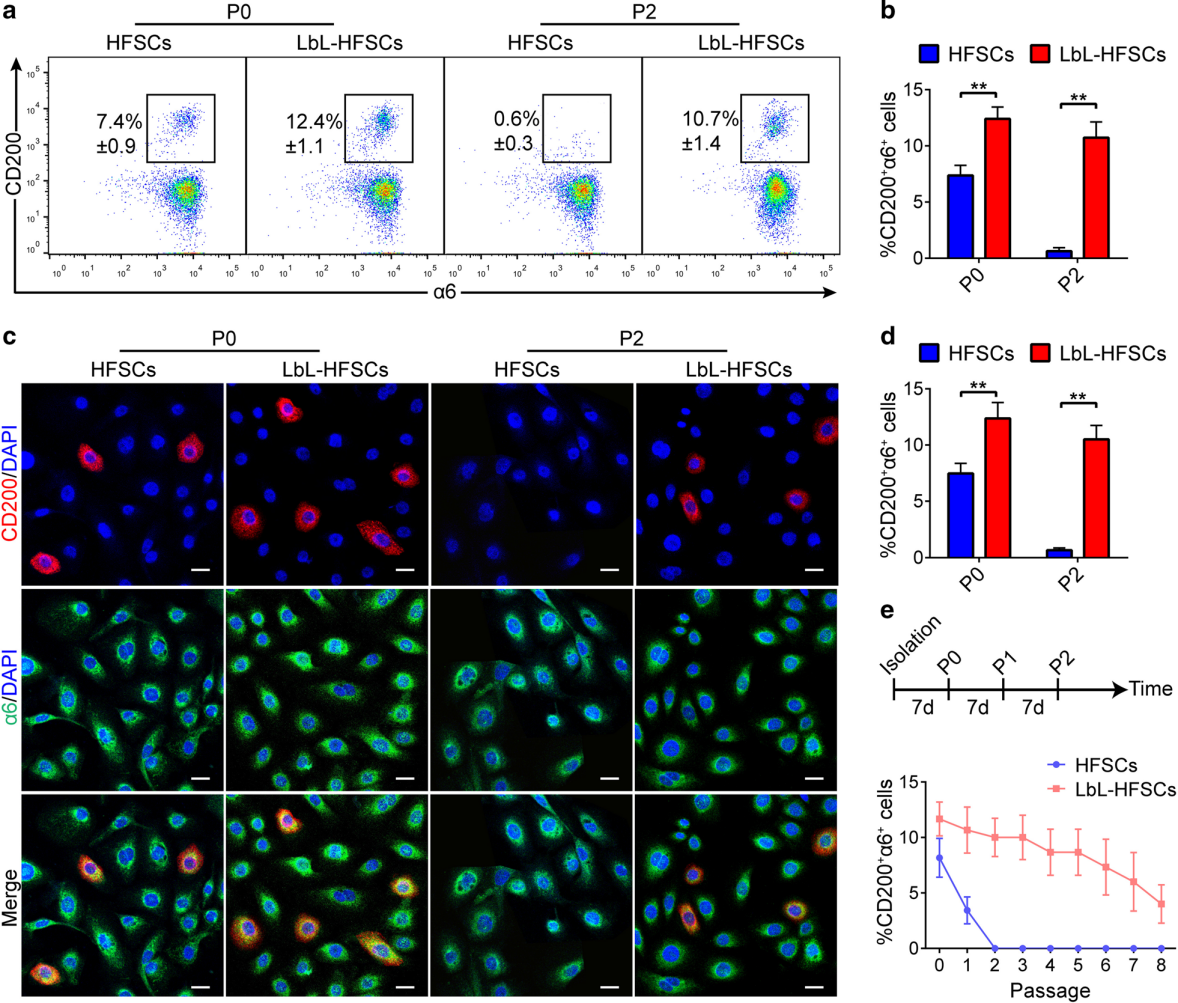
LbL coating maintained HFSC properties. **a**, **b** Flow cytometry was conducted on day 7 after culture. The results showed that the proportion of CD200^+^a6^+^ HFSCs at P0 or P2 in the coated group was higher than the uncoated group (mean ± SD; n = 3; **p < 0.01, Student’s t-test). **c**, **d** Immunofluorescence staining was carried out on day 7 after culture. The proportion of CD200^+^a6^+^ HFSCs at P0 or P2 in the coated group was higher (mean ± SD; n = 4; **p < 0.01, Student’s t-test). DAPI (blue); CD200 (red); a6 (green); scale bars: 20 µm. **e** The long-term cell culture under LbL coating results are from flow cytometry analysis of cells in each passage (mean ± SD; n = 3)

Subsequently, we examined the effect of LbL nanocoating on the properties of human HFSCs. Freshly isolated primary HFSCs were cultured with or without LbL coating, and the LbL-coated group was subsequently coated with LbL during each passage. After 7 days of culture, flow cytometry and immunofluorescence staining were carried out to detect the proportion of CD200^+^α6^+^ HFSCs at P0 and P2 (Figs. 3c and 4a). As shown in Fig. 4b, d, the proportion of CD200^+^α6^+^ cells in the LbL-HFSCs was higher than in the uncoated HFSCs. In the uncoated HFSCs, CD200^+^α6^+^ cells completely disappeared in P2 and could not be further passaged. qRT-PCR and Western blot results were consistent with the above results. The expression of CD200 mRNA and protein of LbL-HFSCs at P0 or P2 was higher than that of uncoated HFSCs (Additional file 1: Figure S2). Besides, after further passage, the LbL-HFSCs maintained the proportion of CD200^+^α6^+^ to some extent (Fig. 4e). These results suggest that the ECM constructed by LbL coating with gelatin/alginate can maintain the CD200^+^α6^+^ HFSC population.

### ***Key growth factors stabilize the CD200***^+^***α6***^+^***HFSC population***

**Fig. 5:**
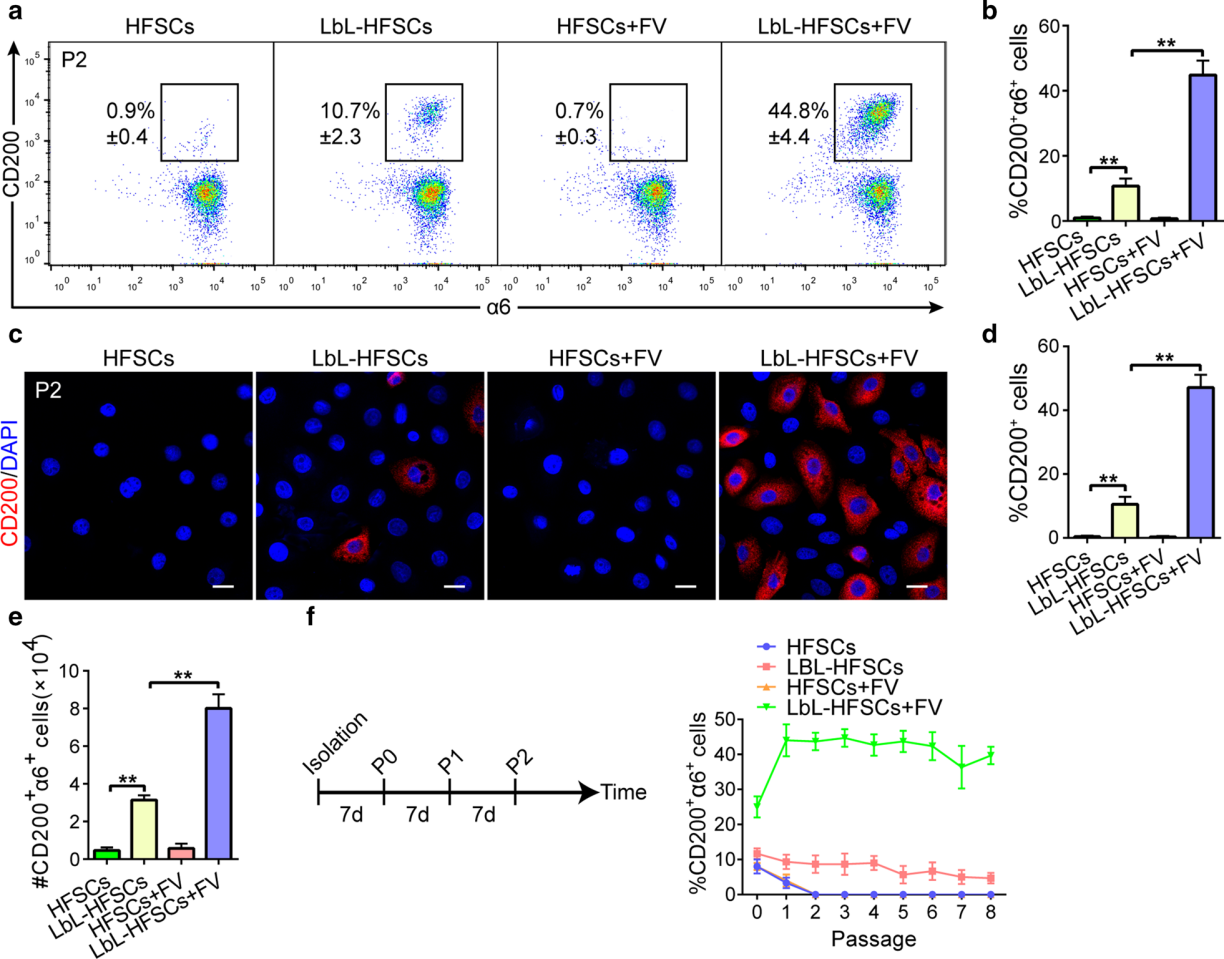
FGF-7 and VEGF-A stabilize the CD200^+^α6^+^ HFSC population. **a**, **b** Flow cytometry conducted on the seventh day after culture at P2. The ratio of CD200^+^a6^+^ HFSCs in the LbL-HFSCs + FV group was higher than the other groups (mean ± SD; n = 3; **p < 0.01, one-way ANOVA). **c**, **d** Immunofluorescence staining performed on the seventh day after culture at P2. Red-stained CD200^+^ HFSCs in the LbL-HFSCs + FV group was higher than the other groups (mean ± SD; n = 4; **p < 0.01, one-way ANOVA). DAPI (blue); CD200 (red); scale bars: 20 µm. **e** Absolute numbers of CD200^+^a6^+^ HFSCs at P2 cultured under different conditions, indicated as fold-change relative to the number of cells inoculated at P0 (mean ± SD; n = 4; **p < 0.01, one-way ANOVA). **f** The results of the long-term cell culture performed under different conditions are from flow cytometry analysis of cells in each passage. Only the LbL-HFSCs + FV culture maintained a stable CD200^+^a6^+^ HFSC population for an extensive period after P2 (mean ± SD; n = 3)

Notably, the CD200^+^α6^+^ HFSC population remained low, regardless of the standard 2D culture conditions or the LbL coating group, which is unsatisfactory for the requirements of further research. Therefore, we aimed to reconstruct the essential components of the HFSC microenvironment in vitro by using the knowledge of signal conduction research in the HFSC microenvironment in vivo. Studies have shown that several growth factors, such as FGF and VEGF-A, which are expressed in hair follicles and regulate their growth [33–35]. After that, we added FGF-7 and VEGF-A to the medium to observe the changes in the CD200^+^α6^+^ HFSC population. Flow cytometry and immunofluorescence staining detected the ratio of CD200^+^α6^+^ HFSC in each group at P2 (Fig. 5a, c). As shown in Fig. 5b, d, the proportion of CD200^+^a6^+^ HFSC population in LbL-HFSCs containing FGF7 and VEGF-A (hereinafter, LbL-HFSCs + FV) was significantly upregulated. Moreover, the absolute number of CD200^+^α6^+^ HFSCs in LbL-HFSCs + FV increased eightfold for cultures at P2, which was much higher than that in the other groups (Fig. 5e). However, even if FGF7 and VEGF-A were added to the uncoated group, the CD200^+^α6^+^ HFSC population still disappeared rapidly. This indicates the importance of the gelatin + alginate ECM construct in maintaining the CD200^+^α6^+^ HFSC population. Surprisingly, the LbL-HFSCs + FV group can be passaged every 7 days. Moreover, the level of CD200^+^α6^+^ HFSCs from P2 remained unchanged (Fig. 5f). Overall, these data indicate that the culture conditions of LbL-HFSCs + FV mimic the conditions of the SC microenvironment in stably maintaining and expanding the 200^+^α6^+^ HFSC population.

### ***LbL coating-sustained release of BMP2 mediates the plasticity of SC to obtain CD200***^+^***α6***^+^***HFSC population***

**Fig. 6:**
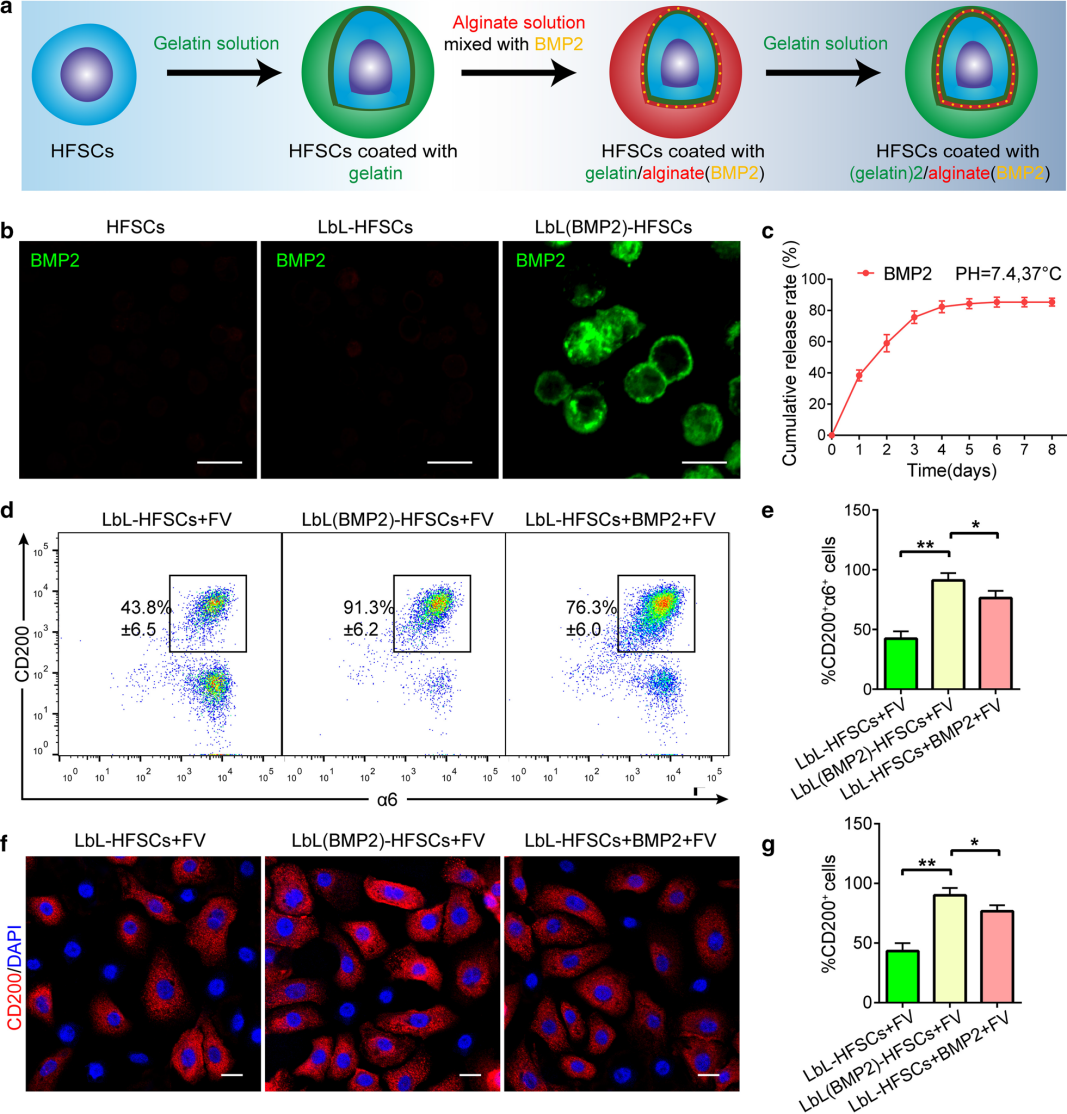
LbL loading BMP2 mediates CD200^+^α6^+^ HFSC population. **a** Schematic diagram of LbL(BMP2)-HFSC construction. **b** Immunofluorescence staining against BMP2, confirming the presence of BMP2 on the cell surface of LbL(BMP2)-HFSCs only. BMP2 (green), scale: 20 μm. **c** The release profile of BMP2 conducted using enzyme-linked immunosorbent assay (ELISA) at different time points after coating (mean ± SD; n = 4). **d**, **e** Flow cytometry performed on the seventh day after P2 culture. The proportion of CD200^+^a6^+^ HFSCs in the LbL(BMP2)-HFSCs + FV group was higher than the other groups (mean ± SD; n = 3; *p < 0.05, **p < 0.01, one-way ANOVA). **f**, **g** Immunofluorescence staining performed on the seventh day after P2 culture. Number of red-stained CD200^+^ HFSCs in the LbL(BMP2)-HFSCs + FV group was higher than the other groups (mean ± SD; n = 4; *p < 0.05, **p < 0.01, one-way ANOVA). DAPI (blue); CD200 (red); Scale bars: 20 µm

Subsequently, we tried applying known signaling pathways that mediate the transition between HFSCs and their progeny to obtain more purified CD200^+^α6^+^ HFSCs. BMP signaling was confirmed to maintain the properties of HFSCs by inhibiting their differentiation. We hypothesized that additional BMP2 would prevent CD200^+^α6^+^ HFSCs from generating differentiated progeny, thereby increasing the ratio of CD200^+^α6^+^ HFSCs further. First, we loaded the bioactive molecule BMP2 into the LbL nanocoated layer as a sustained release drug carrier to regulate the fate of the human HFSCs, which is essential for evaluating the potential application of LbL technology in SC microenvironment construction. After coating the cells with the first layer of gelatin, we loaded BMP2 into the alginate layer and coated the HFSCs. We then coated the HFSCs with gelatin to obtain LbL(BMP2)-HFSCs (Fig. 6a). To show BMP2 on the cell surface, we incubated each group with anti-BMP2 antibodies. Data showed that only LbL(BMP2)-HFSCs displayed positive results (Fig. 6b). In a physiological environment (pH = 7.4, T = 37 °C), LbL(BMP2)-HFSCs exhibits a time-varying release characteristic for about 7 days (Fig. 6c). Flow cytometry and immunofluorescence staining detected the ratio of CD200^+^α6^+^ HFSC in each group at P2 (Figs. 5f and 6d). The results showed that the proportion of CD200^+^α6^+^ HFSC population in LbL(BMP2)-HFSCs + FV was higher than that of LbL-HFSCs + FV and LbL-HFSCs + BMP2 + FV (Fig. 6e, g). Collectively, these results demonstrate that compared with the direct addition of BMP2 to the culture medium, the loading of BMP2 can promote the acquisition of the CD200^+^α6^+^ HFSC population. Besides, the absolute number of CD200^+^α6^+^ HFSC cells in the LbL(BMP2)-HFSCs + FV increased by a factor of eight when cultured to P2 (Additional file 1: Figure S3A). The ratio of CD200^+^α6^+^ HFSC cells in the LbL(BMP2)-HFSCs + FV remained stable, and could be passaged and expanded for prolonged period (Additional file 1: Figure S3B). These findings confirm that the loading of BMP2 regulates the fate of human HFSCs more effectively than directly adding BMP2 to obtain a purer CD200^+^α6^+^ HFSC population.

## Discussion

A fundamental issue in the field of human hair follicle engineering and SC microenvironment research is the construction of a culture model for the stable expansion of human HFSCs and regulation of their fate in vitro. Consistent with previous studies, our results indicate that remodeling the microenvironment of HFSCs in vitro could maintain the property and plasticity of HFSCs, which is lost under standard 2D culture conditions.

Thus, this study reconstructed a nanoscale biomimetic microenvironment for expanding SCs and revealing their plasticity using LbL self-assembly. As a simple, reliable, efficient, and versatile method, LbL self-assembly technology has been widely used to design and fabricate various nanostructured coatings, films, and scaffolds with tailored physicochemical properties and tunable architectures [25]. This technology has successfully encapsulated and constructed an ECM for various cells, including pancreatic beta cells and neural SCs, thereby promoting their cell function [26, 36]. Here, we employed LbL self-assembly using gelatin and alginate to construct a nanoscale ECM for a single human HFSC. LbL has unique advantages as a technology for constructing a biomimetic ECM for cells at the single-cell level. Our results demonstrate that LbL nanocoating using gelatin and alginate can be applied uniformly in all cells without damaging the HFSC, and helps maintain their SC properties to a certain extent. Additionally, the results of SEM analysis indicated that LbL-HFSC presented a *vivo*-like 3D structure. This suggests that the LbL nanocoating can provide mechanical support for human HFSCs, keeping them sparsely distributed in a spherical state, alike the in vivo cell microenvironment.

ECM not only regulates cell behavior but also serves as a cell scaffold. Moreover, it plays an essential role in organ development, function, and repair [31]. Some studies have shown that matrix stiffness and its area, rather than ECM composition or concentration, are crucial for the differentiation of keratinocytes, which suggests that biophysical factors are more important in determining the fate of SCs [37]. Moreover, changes in ECM mechanics and cell shape may be transmitted to the nucleus and regulate gene transcription programs [38]. These studies were consistent with our results; the LbL-coating using gelatin/alginate can provide a biomimetic ECM and mechanical support for HFSCs, maintaining their SC properties.

Although the biomimetic ECM composed of gelatin and alginate can maintain the properties of HFSCs to a certain extent, the proportion of CD200^+^α6^+^ HFSCs population remains minimal and gradually declines. This indicates that only the coating layer constructed by gelatin and alginate did not satisfy the basic requirements of the SC microenvironment. Studies have shown that besides extracellular proteins, key growth factors are critical for constructing the SC microenvironment [10]. Therefore, we added FGF7 and VEGF2 to the culture medium. Resultantly, the proportion of the CD200^+^α6^+^ HFSC population was significantly increased and remained stable after P2, while human HFSCs stably expanded on a large scale. Thus, our results showed that the minimal microenvironment requirement of HFSCs involved ECM and key growth factors.

Lineage tracing and ablation studies have shown that HFSCs are essential for hair regeneration, and the activated progeny refill the ablated SC chamber to sustain hair regeneration [24, 39]. This indicates the plasticity of HFSCs and their progeny in vivo. In other words, the population of CD200^+^α6^+^ HFSCs in the bulge area can be re-populated by the CD200^−^α6 + HFSCs in the non-bulge area. Studies have also confirmed that the plasticity of mouse HFSCs is regulated by BMP and Shh signals in vitro. This study used the LbL nanocoating as a sustained-release drug carrier and loaded the active factor-BMP2 to further regulate the fate of HFSCs to reveal the plasticity of human HFSCs in vitro. Our results showed that the proportion of CD200^+^α6^+^ HFSCs increased significantly after loading BMP2, reaching 90%. This lays the foundation for the expansion of specific types of purified HFSCs. Besides, by directly separating the ORSCs for culturing, and regulating their fate to obtain a purified population of CD200^+^α6^+^ HFSCs, the expansion efficiency and use of HFSCs are highly improved. This avoids the waste of precious human hair follicle tissue amplified by CD200^+^α6^+^ HFSCs and purified by FACS, as occurred in previous studies.

Using our innovative biomimetic culture system, we successfully constructed the minimum necessary components for the microenvironment of human HFSCs. This method can not only stably expand human HFSCs but also regulate their fate to reveal their plasticity. This culture system provides an important research avenue for human hair follicle engineering and SC microenvironment research.

## Conclusions

Studies on good culture models that can steadily augment human HFSCs on a large scale with control remain of growing interest in tissue hair follicle engineering and SC homeostasis. Here, we constructed a 2D biomimetic culture system using LbL self-assembly with gelatin and alginate. The system included a biomimetic ECM and some key signal molecules to minimize the niche of simulated SCs, which effectively and stably expanded HFSCs. Loading BMP2 to the LbL nanocoated layer can regulate the plasticity of human HFSCs to obtain purified CD200^+^α6^+^ HFSC populations. This system can simulate the microenvironment of HFSCs more reasonably and comprehensively, thus stably amplifying HFSCs and revealing their plasticity, providing a novel method for hair follicle reconstruction and SC microenvironment research.

## Methods

### Cell cultures

With the approval of the Medical Ethical Committee of Southern Medical University, after obtaining informed consent, the human hair follicles were obtained from the hair transplantation surgery by follicular unit extraction (FUE). Single HF was dissected and separated from the follicular unit using microforceps under a stereoscope. The lower part (hair papilla area) and the upper part (above the sebaceous glands) of the hair follicle were removed, and the remaining hair follicle tissue was digested with 0.1% dispersed TICs (theca-interstitial cells) preparations and intact follicles (Invitrogen Biotech, Carlsbad, CA, USA) for 50 min and shaken every 15 min at 37 °C. Then the dermal sheath under the stereoscope was removed and the remaining hair follicles containing the outer root sheath were digested by trypsin (0.05%; Gibco, Gaithersburg, Maryland, USA) for 10–15 min at 37 °C. To terminate digestion, the same amount of DMEM containing 10% fetal bovine serum (FBS; Gibco) was added and the cells were filtered with a 70 μm filter (Corning, New York, USA). The cell filtrate was subsequently centrifuged at 350×*g* for 5 min, and then washed twice with DMEM. For 2D culture of human HFSCs, collagen I (30 μg/mL; Millipore) and fibronectin (10 μg/mL; Millipore) in a T25 culture flask were coated in advance at 37 °C for 1 h, then the cells were re-suspended in KGM medium and inoculated on a T25 culture flask. For the LbL culture of human HFSCs, HFSCs were coated with gelatin and alginate, and the cells were re-suspended in KGM medium and cultured in a T25 culture flask pre-coated with Geltrex (Gibco).

### Flow cytometry

The cell suspension was washed twice in KGM medium and stained with 10 μL fluorescent labeled antibody APC-CD200 (Abcam; Cambridge, MA, USA) at 4 °C and PE-a6 integrin (Abcam) for 20 min. HFSCs cell suspension was washed twice with buffer and analyze under LSRFortessa (BD Biosciences, San Jose, CA, USA). Use FlowJo software version 10 (BD Biosciences) for subsequent data analysis.

### LbL coating of single HFSC

The human HFSCs (2 × 106) cell suspension was transferred into 15 mL tube and centrifuged at 350×*g* for 5 min. Then, 0.1% gelatin (2 mL; Thermo Fisher Scientific, Waltham, Massachusetts, USA) was added to the supernatant, mixed thoroughly and incubated on ice for about 10 min with repeated shaking. The supernatant was removed after centrifugation at 350×*g* centrifugation for 5 min. Subsequently, the cell precipitate was washed twice with DPBS (5 mL; Gibco) and 0.1% alginate (2 mL; Thermo Fisher Scientific) solution was then added. As mentioned above, the HFSCs were incubated for another 10 min. The supernatant was removed after centrifugation for 5 min at 350×*g*. The entire process was repeated to add another layer of gelatin on HFSCs, and finally, the HFSCs were coated with three layers of biomimetic material.

### Preparation of fluorescence-labeled materials

Commercial FITC-gelatin was acquired from Invitrogen. The preparation of rhodamine B conjugated-alginate was as follows. First, rhodamine B (20 mg; Invitrogen) added to 2 mL DPBS; then, 1-ethyl-3-(3 (dimethylamino) propyl) carbodiimide (EDC; 40 mg; Thermo Fisher Scientific) and 1-hydroxy-2-line 5-pyrrolidine dione (NHS; 1.75 mg; Thermo Fisher Scientific) were added; the sample was incubated at room temperature (25 ± 1 °C) for 30 min. Ethylenediamine was subsequently added and the sample incubated for 12 h. After dialysis and freeze-drying, rhodamine B-ethylenediamine powder was dissolved in 1% alginate solution (2 mL) and adding EDC (10 mg). The solution was oscillated overnight at room temperature, dialyzed, and freeze-dried to obtain rhodamine B-alginate powder.

### Scanning electron microscopy (SEM)

The samples were fixed using 2.5% glutaraldehyde (1 mL), and then dehydrated in ethanol. After platinum coating, JSM-6330F (JEOL, Tokyo, Japan) was used to characterize HFSCs and LbL-HFSCs.

### Zeta-potential assessment

We separately collected the uncoated HFSCs, HFSCs coated by gelatin, HFSCs coated by gelatin/alginate, or the HFSCs coated by (gelatin) 2/alginate. A Zetasizer Nano ZS (Malvern Instruments, Malvern, England) was used to determine the zeta potentials of the four groups.

### Live/dead staining

According to the reagent instructions, all samples were stained for 15 min using living/dead cell-staining (Invitrogen). A fluorescence microscope (IX71 FL, Olympus, Tokyo, Japan) was used to visualize images.

### Cloning formation assay

HFSCs/well (1 × 10^3^ cells/well) were cultured for 14 days in 6-well plates pre-coated with a mixture of collagen I and fibronectin, or Geltrex. Colonies were then stained with Giemsa Stain and counted for their colony number.

### Immunofluorescence

After washing once with DPBS, the cells were fixed with 4% paraformaldehyde at room temperature for 15 min, and 0.3% Triton X was permeated for 5 min. After blocking the samples for 30 min with 5% BSA, the samples were incubated with the primary antibody against CD200 (1:200, Abcam), a6 (1:100, Abcam) or BMP2 (1:100, Abcam) overnight at 4 °C. The cells were stained with fluorescent secondary antibody at room temperature for 1 h and DPAI for 5 min. Fluorescence microscope images were obtained using CLSM (LSM880, Cari Zeiss, Jena, Germany).

### BMP2 loading and ELISA

For the loading of BMP2 (Abcam), we followed the same procedure as that for LbL cell coating, except tha**t** 100 ng/mL BMP2 was added to 1 mL 0.1% alginate and loaded into the second nanocoated layer. For ELISA, samples (20 mL) obtained at specified time points were added to a microwell plate pre-coated with anti- BMP2 for 2 h. Subsequently, each well was added to the conjugated solution for 1.5 h, and the solution was added to the culture plate for 25 min. After that, the termination solution added. A multi-label counter was used to measure absorbance at 450 nm.

### Quantitative real-time polymerase chain reaction (qRT-PCR)

Total RNA was extracted from HFSCs with TRIzol (Invitrogen) and then reverse transcribed with PrimeScript® RT reagent (Takara Bio, Shiga, Japan). Using the Stratagene MX3005P qRT-PCR system (Agilent Technologies, Santa Clara, CA, USA), we followed the manufacturer’s operating procedures for qRT-PCR. The primer sequence is listed in Additional file 1: Table S1.

### Western blotting

Proteins were extracted using a lysis buffer and quantified using a BCA Protein Assay Kit (Thermo Fisher Scientific, West Palm Beach, FL). The protein lysate was electrophoresed using 10% SDS-PAGE and transferred to a PVDF membrane (Roche). The membranes were incubated with primary antibodies CD200 (1:1,000, Abcam) at 4 °C overnight. After washing, secondary antibodies were labeled with HRP, and the signals were photographed. The band intensity of the protein was determined by densitometry using ImageJ software. GAPDH served as the loading control.

### Statistical analysis

Statistical analyses were conducted using SPSS software (SPSS, version 18.0) or GraphPad Prism software (GraphPad, version 7.0). Statistical significance was determined using the Student’s t-test or the one-way analysis of variance (ANOVA). The data were expressed as mean ± standard deviation (SD). Each experiment was performed at least three times. p < 0.05 was considered significant.

## Supplementary Information

**Additional file 1: Table S1.** Primers used for quantitative real-time polymerase chain reaction. **Figure S1.** Colony formation assay analysis of cell proliferation. **Figure S2.** SC properties of LbL-HFSCs. **Figure S3.** Long-term culture of HFSCs under different conditions.

## Data Availability

All data and materials are included in the manuscript.

## Abbreviations

ECM: Extracellular matrix
FUE: Follicular unit extraction
FACS: Fluorescence-activated cell sorting
HFSCs: Hair follicle stem cells
KGM: Keratinocyte growth medium
LbL: Layer-by-layer
ORSCs: Outer root sheath cells
SCs: Stem cells
SEM: Scanning electron microscopy
qRT-PCR: Quantitative real-time polymerase chain reaction
