# Supplementary material for: Nanoscale microenvironment engineering for expanding human hair follicle stem cell and revealing their plasticity
Source: J Nanobiotechnology. 2021 Mar 31;19:94. doi: 10.1186/s12951-021-00840-5 (PMC8010974; doi:10.1186/s12951-021-00840-5)
Supplement: Supplementary file 1 — Additional file 1: Table S1. Primers used for quantitative real-time polymerase chain reaction. Figure S1. Colony formation assay analysis of cell proliferation. Figure S2. SC properties of LbL-HFSCs. Figure S3. Long-term culture of HFSCs under different conditions. [file 12951_2021_840_MOESM1_ESM.docx]

**Additional Material**

**I. Additional Tables**

**Table S1.** Primers used for quantitative real-time polymerase chain reaction

| Gene name | Sequences (5' to 3') | |
| --- | --- | --- |
|  | Forward primer | Reverse primer |
| *CD200* | AAGTGGTGACCCAGGATGAAA | AGGTGATGGTTGAGTTTTGGAG |

**II. Additional Figures and Legends**

**
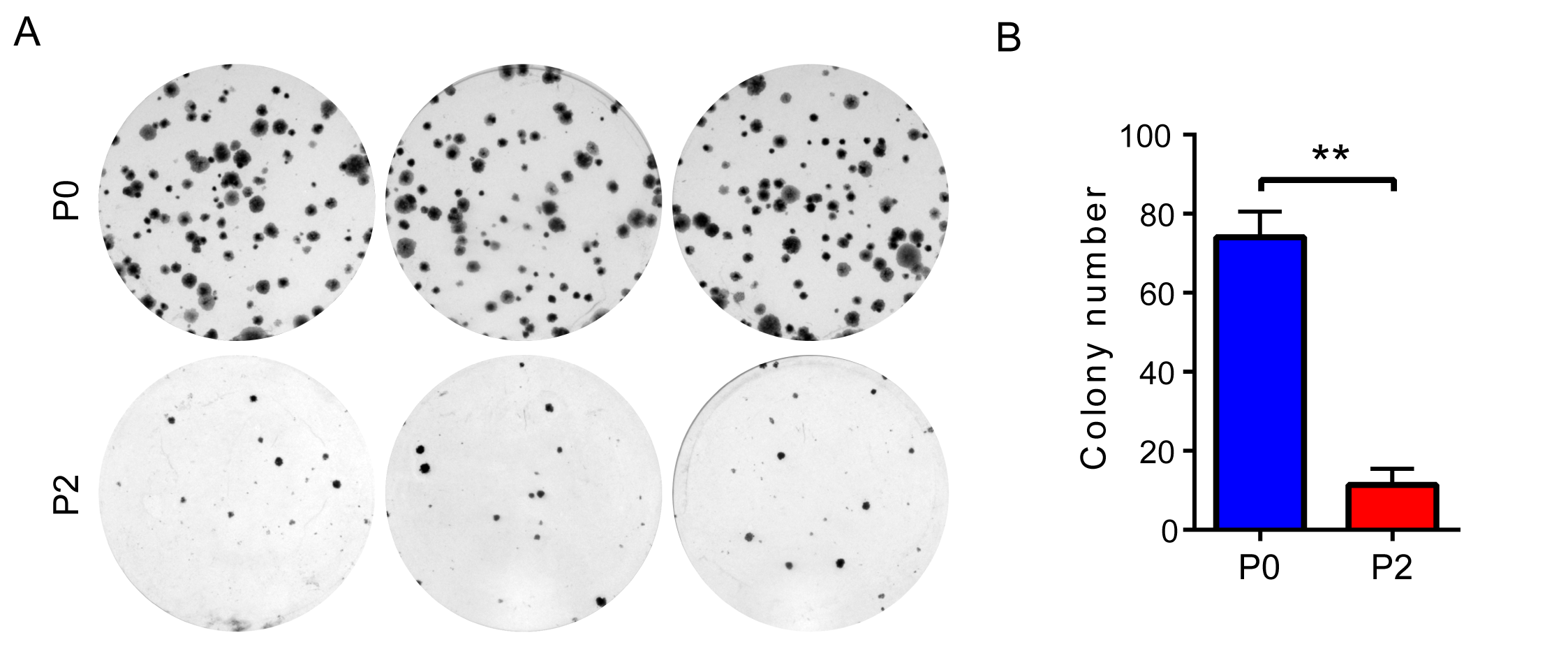
**

**Figure S1.** **Colony formation assay analysis of cell proliferation.** (A) Colony formation assay was used to detect the cell proliferation of HFSCs at P0 and P2. (B) The proliferation of HFSCs was significantly down-regulated after culture (mean ± SD; n = 4; ***p* < 0.01, Student’s t-test).


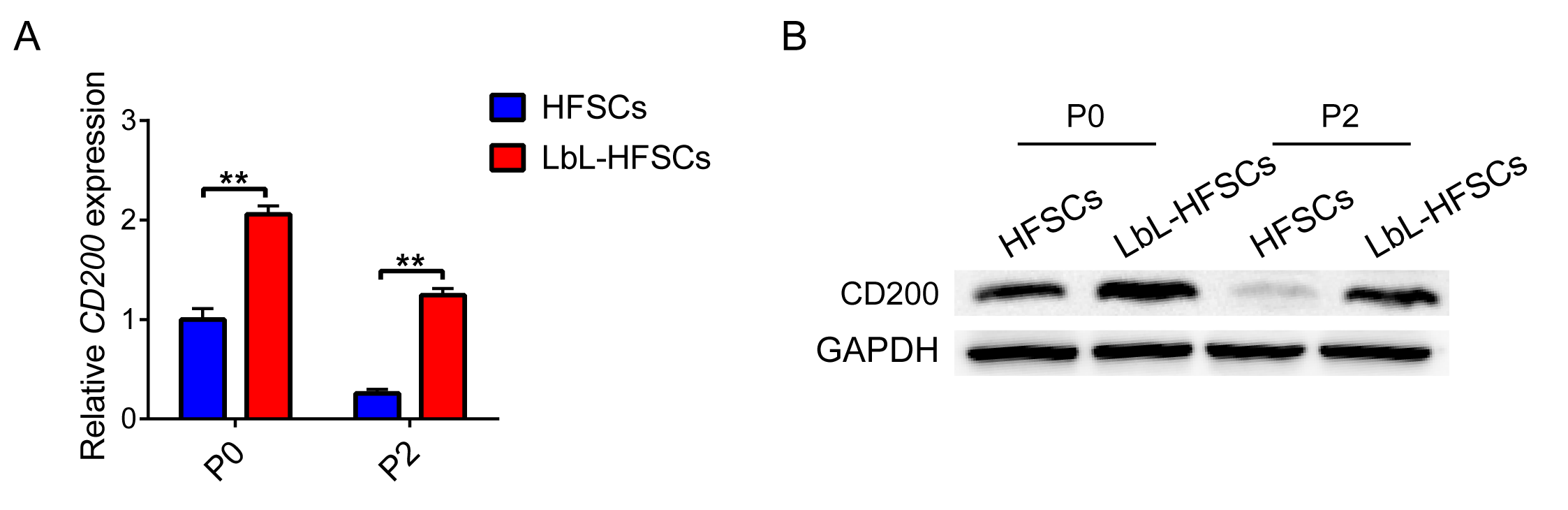


**Figure S2.** **SC properties of LbL-HFSCs.** (A) Quantitative reverse transcription-polymerase chain reaction (qRT-PCR) analysis of *CD200* mRNA expression. In P0 or P2, the expression of *CD200* in LbL-HFSCs was significantly higher than in HFSCs (mean ± SD; n = 3; ***p* < 0.01, Student’s t-test). (B) Western blotting of CD200 protein expression. LbL-HFSCs exhibited higher expression of CD200 than HFSCs in P0 or P2. **p* < 0.05; ***p* < 0.01.

**
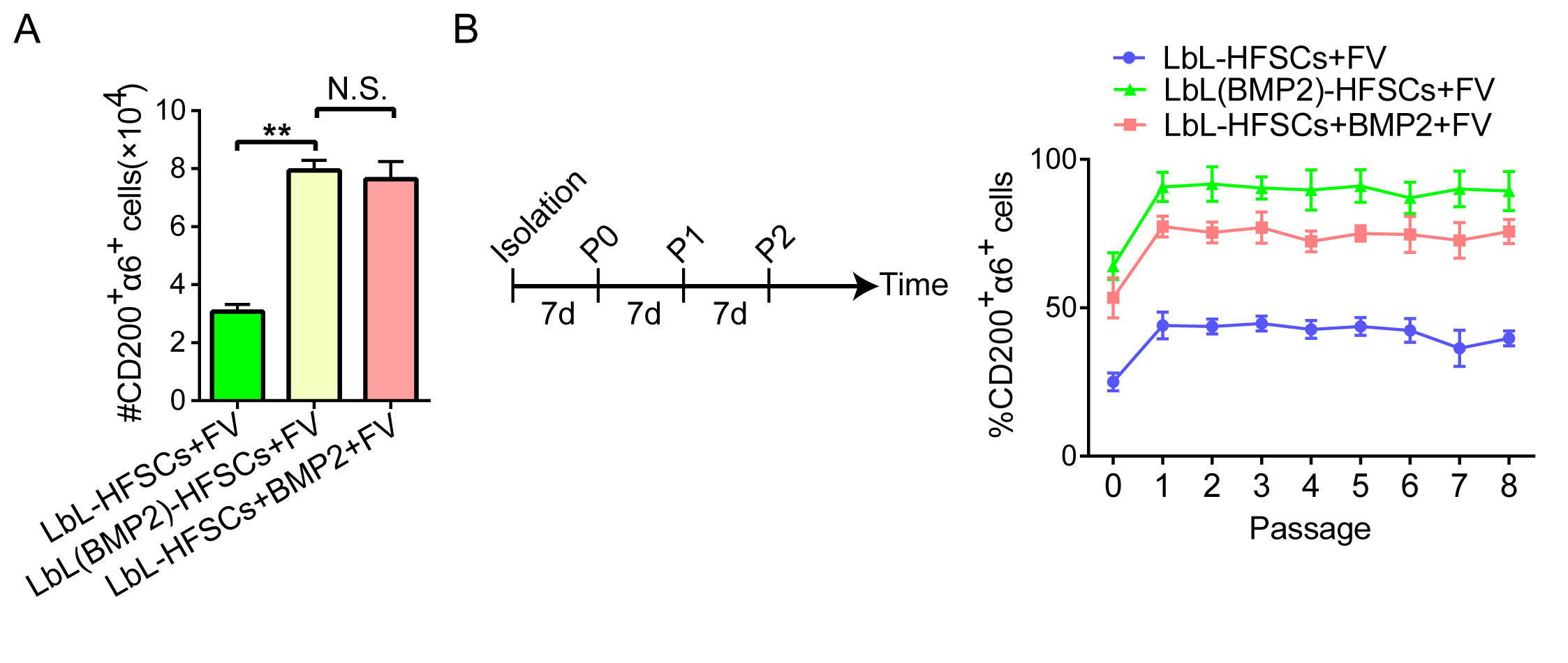
**

**Figure S3.** **Long-term culture of HFSCs under different conditions. (A)** Absolute numbers of CD200^+^a6^+^ HFSCs at P2 cultured under different conditions. The results are shown as the fold-change relative to the number of cells inoculated at P0 (mean ± SD; n = 4; NS, not significant; *p* > 0.05, ***p* < 0.01, one-way ANOVA). **(B)** Each culture condition can maintain a stable CD200^+^a6^+^ HFSC population for a long time after P2. The results are from flow cytometry analysis of cells in each passage. LbL (BMP2) + FV had a higher proportion of CD200^+^a6^+^ HFSCs (mean ± SD; n = 3).
